# Evaluation of patient-centered rehabilitation model targeting older persons with a hip fracture, including those with cognitive impairment

**DOI:** 10.1186/1471-2318-13-136

**Published:** 2013-12-13

**Authors:** Katherine S McGilton, Aileen M Davis, Gary Naglie, Nizar Mahomed, John Flannery, Susan Jaglal, Cheryl Cott, Steven Stewart

**Affiliations:** 1 Department of Research, Toronto Rehabilitation Institute, E.W. Bickle Centre for Complex Continuing Care, 130 Dunn Avenue, Toronto, ON M6K 2R7, Canada; 2 Lawrence S. Bloomberg Faculty of Nursing, University of Toronto, 155 College Street, Toronto, ON M5T1P8, Canada; 3 Division of Health Care and Outcomes Research, Toronto Western Research Institute, 399 Bathurst Street, Toronto, ON M5T 2S8, Canada; 4 Department of Medicine, Baycrest Geriatric Health Care Centre, 3560 Bathurst Street, Toronto, ON M6A 2E1, Canada; 5 MSK Rehabilitation Program, Toronto Rehabilitation Institute, University Centre, 550 University Ave, Toronto, ON M5G 2A2, Canada; 6 Department of Physical Therapy, University of Toronto, 160-500 University Avenue, Toronto, ON M5G 1V7, Canada; 7 School of Kinesiology and Health Studies, Queen’s University, 28 Division St., Kingston, ON K7L 7N9, Canada

**Keywords:** Person-centered care, Cognitive impairment, Rehabilitation, Healthcare restructuring, Hip fracture

## Abstract

**Background:**

The purpose of this study was to evaluate outcomes for older persons post-hip fracture repair, including those with cognitive impairment (CI), following implementation of a novel model of care – the Patient-Centered Rehabilitation Model including persons with CI (PCRM-CI). The PCRM-CI is an interdisciplinary rehabilitation program that incorporates education for healthcare professionals (HCPs), including nurses, which is focused on geriatric care including management of dementia and delirium, support for HCPs from an Advanced Practice Nurse, and family support and education. Primary outcome measures were mobility gain from admission to discharge and whether or not patients returned home post-discharge.

**Methods:**

The PCRM-CI intervention was evaluated using a quasi-experimental design, following implementation in two community hospital inpatient rehabilitation units. One hundred forty-nine patients aged 65 and older participated as patients in the usual care (76) or PCRM-CI intervention (73) groups. Patient mobility was assessed at admission and discharge by the Functional Independence Measure Motor Subscale (FIMM); the difference in mobility scores was defined as mobility gain. Patient discharge location was also captured to determine whether or not patients returned home from inpatient rehabilitation.

**Results:**

No difference in mobility gain was found between the usual care and PCRM-CI groups as measured by the FIMM. Patients in the intervention group were more likely to return home post-discharge than those in the usual care group (*p* = 0.02).

**Conclusions:**

Results of the PCRM-CI evaluation suggest that older adults with CI can successfully be rehabilitated post-hip fracture repair using this novel, interdisciplinary rehabilitation program.

**Trial registration:**

This trial has been registered with the US National Institutes of Health (ID: NCT01566136)

## Background

Despite improvements in surgical repair of hip fractures, the path of recovery is fraught with medical, social, and economic consequences that result in significant impairment in mobility and function for many elderly persons. Recovery may also be complicated by delirium and/or dementia [1] characterized by the presence of cognitive impairment (CI) across a spectrum of severity. Research has indicated that 31% to 65% of older persons with hip fracture have CI [2]; therefore, rehabilitation for these patients becomes more complex.

In recognition of this complexity, specialized programs have been developed to improve outcomes for older persons post-hip fracture. Acute care interventions implemented on orthopedic wards have led to fewer medical complications and lower rates of mortality [3-7]. Evidence of positive functional gain, reduced length of stay, and fewer complications post-hip fracture in older persons with CI has emerged over the past 15 years from specialized geriatric rehabilitation units that admit patients with CI [8-12]. However, with the aging population and the increase in number of persons with CI, insufficient specialized geriatric rehabilitation beds are available for older persons post-hip fracture [13] but there are no plans to create more of these units. Moreover, people with CI are often refused entry into general musculoskeletal (MSK) inpatient rehabilitation programs as staff believe they cannot be rehabilitated. For those few patients post-hip fracture with CI transferred to general MSK inpatient rehabilitation units, the professional healthcare providers (HCPs) there often lack the necessary skills and knowledge about cognitive and behavioral strategies to provide adequate care for these complex patients [14,15]. In order to accommodate patients post-hip fracture with CI in general MSK inpatient rehabilitation units, HCPs may be required to develop new competencies to care for this growing population.

Responding to this pressing need, our team developed a patient-centered rehabilitation model of care for all older patients with hip fracture that incorporates specific components targeting patients with CI (PCRM-CI). To our knowledge, no other model of care for general MSK rehabilitation programs combines an intensive rehabilitation program for older persons post-hip fracture (i.e., up to two hours of rehabilitation per day) with delirium and dementia management, while providing concurrent support for HCPs and family members. The model relies on the provision of training and education for existing HCPs to learn how to care for older persons post-hip fracture with CI and multiple comorbidities with support from an Advanced Practice Nurse (APN) with gerontological expertise.

The main objectives of the current study were to determine whether using the PCRM-CI on MSK rehabilitation units could increase patient mobility and the probability of returning home (i.e., return to prior residence, including family home or retirement home, and excluding nursing home) for older adults post-hip fracture compared to usual care. We hypothesized that the PCRM-CI would result in a) increased patient mobility and b) greater likelihood of returning to pre-fracture residence as compared to usual care.

## Methods

### Study design

A quasi-experimental design was used to evaluate the PCRM-CI. Comparisons were made between two groups of inpatients undergoing MSK rehabilitation post-hip fracture: those receiving usual care (control group) and those receiving the PCRM-CI intervention (intervention group).

### Setting

Participants for both groups were recruited from two Ontario Community Hospitals: Site I, a 40-bed unit in a 500-bed hospital, and Site II, a 20-bed unit in a 120-bed hospital.

### Eligibility

Patients were eligible to participate if they were: (a) 65 years or older; (b) with or without CI; (c) admitted to rehabilitation directly from acute care after receiving surgery for hip fracture; (d) living at home prior to the hip fracture (i.e., living in a family home or retirement home); and (e) had a family member or close friend who could act as collateral informant.

### Groups

#### Usual care

Usual care consisted of rehabilitation management, including physical assessments at admission, and daily one-hour sessions of physical and occupational therapy, five days per week (e.g., hip range of motion, lower extremity strengthening, daily increase of ambulation distance using the lowest level of assistance/aids required, and increasing independence in basic activities of daily living). Usual care did not include screening of patients for dementia or delirium.

#### PCRM-CI intervention

The PCRM-CI included five main components: (a) rehabilitation management (as described above); (b) dementia management (if required); (c) delirium prevention; (d) education and support for the HCPs on providing patient-centered care for older persons with multiple comorbidities including CI; and (e) support and education for family caregivers, which included best strategies to keep their family members mobile and independent. During the first week of admission, patient goals were developed with input from family caregivers; a target discharge date was discussed and set amongst the patient, family, and staff.

A newly developed clinical pathway – the PCRM-CI – was provided to guide staff through program delivery. As part of the implementation, an APN with expertise in gerontology, and fully trained in the PCRM-CI, was present on each of the units for one year to assist staff in learning to conduct bedside assessments and interventions and to supervise the care they provided. Staff training included instruction for use of the Confusion Assessment Method (CAM) on admission, a focus on prevention strategies, and when there were any changes in the patient’s cognition, specific interventions to manage delirium. Additional point-of-care in-services were provided as required, which focused on non-pharmacological sleep interventions, pain management, and bladder re-training. For dementia management, the APN introduced the REAP model, a person-centered approach to care for complex older adults that helps compensate for a patient’s loss of cognitive reserve [16]. The REAP model has four components: (a) *Relate well*, which equips staff with interactional strategies and techniques to compensate for a patient’s loss of cognitive function (e.g., using one step commands if necessary) [17]; (b) modification of the *Environment*[18] to accommodate and enable a patient’s changing cognitive abilities (e.g., HCPs are taught to control the daily activity schedule so as not to over- or under-stimulate the patient); (c) emphasis on *Abilities-focused* care [19], during which HCPs focus on a patient’s retained abilities and compensate when necessary (e.g., the ability to initiate activities may be threatened because of CI, so HCPs are taught to follow the steps of asking, cueing, and demonstrating before doing the activity for the patient); and (d) the concept of *Personhood*[20], which refers to gaining more knowledge of a person’s life to motivate the person during rehabilitation and care provision.

### Enrollment and implementation

Participants were enrolled in the study in two phases: between January 2009 and June 2010, all eligible, consenting patients admitted consecutively at both sites were enrolled in the usual care group. Following staff consent and a workshop, the PCRM-CI model was then implemented at both sites. Recruitment to the PCRM-CI intervention groups occurred between August 2010 and March 2012. Ethics approval was obtained from the Research Ethics Board at both sites and from Toronto Rehabilitation Institute. Written informed consent was obtained from both the patient and the collateral informant at the first meeting following admission. The study is registered at http://clinicaltrials.gov/show/NCT01566136.

Following enrollment, participants in both groups underwent a series of standardized clinical and functional assessments within 24 hours of admission. Data were obtained from personal interviews, collateral informants, and medical records. A Research Assistant at each site, who was not involved with patient care, conducted all interviews. Baseline (within 24 hours of admission) information included: patient demographics (i.e., age, sex, living situation [living alone or with someone], and comorbid conditions); cognitive status according to the Mini-Mental State Examination (MMSE) [21] (a score of 23 or less indicates the presence of CI); pre-fracture cognitive decline, assessed by the Informant Questionnaire on Cognitive Decline in the Elderly (IQCODE), which measures memory before hip fracture compared to 10 years earlier [22]; indication of delirium, according to the CAM, which was collected weekly [23]; and pre-fracture functional status, according to the Older Americans Resources and Services Instrument (OARS) [24].

The primary outcome for this study – mobility – was measured using the Functional Independence Measure Motor Subscale (FIMM). This scale includes assessments of both locomotion and mobility and measures independence in walking, climbing stairs, doing bed transfers and transfers from the toilet and tub/shower [25]. The FIMM has demonstrated reliability, validity, and responsiveness for detecting improvement in mobility independence for patients with hip fracture [26]. The secondary outcome was the proportion of participants that returned home after discharge from inpatient rehabilitation post-hip fracture repair. These data were collected at admission and at discharge.

#### PCRM-CI intervention strategy

Implementing a new model of care involves negotiating and developing shared understandings about the beliefs, risks, and advantages of the new over the old approach [27]. A one-day workshop was delivered to all staff providing care on the unit. Given the potential of staff to feel threatened by this change, all efforts were made to make the workshop interactive. The workshop was run by the study’s Principal Investigator (PI) and the APN with the purpose of enhancing knowledge and challenging the attitudes and beliefs held by staff regarding caring for persons with CI. Staff were invited to identify any perceived risks from their perspective; some expressed concern about caring for patients with CI, especially regarding behaviors that such patients could exhibit. To address staff concerns, a gradual admission of post-hip fracture patients with CI was implemented (i.e., only one post-hip fracture patient with CI was admitted to the unit at the start of the study). Once staff felt comfortable caring for persons with CI, the number of such post-hip fracture patients increased until the units reached capacity.

Perhaps the most useful strategy employed during the workshop was involving HCPs from the initial pilot site where the PCRM-CI model of care was first introduced and preliminary evidence of the model’s effect was found [28]. Staff from that site discussed their first-hand experiences of patients with CI who had been successfully rehabilitated when staff used the components of the REAP model within the context of the overall approach to care in the PCRM-CI model. They also admitted their pre-existing fears and disbelief about being able to rehabilitate this population and explained how, over time and with the help of the REAP model, their preconceived ideas were transformed into a more positive and accepting perspective.

Despite positive feedback from staff about the workshop, education alone may not change practice, as it does not close the gap between knowing and doing [29]. To increase the likelihood of successful implementation of the PCRM-CI, the APN acted as a facilitator to help staff understand the importance of making a change, what was needed to change, and the process for effectively making the change. In order to do so, the APN had to become familiar with the context which consists of the dimensions of culture, leadership, and evaluation [27]. The following strategies were put in place to assist with understanding and then enhancing the context during the implementation phase. First, the APN took time to understand the culture of each unit (i.e., the management styles of the charge nurses and unit managers), the work environment and workloads, and the knowledge, skills, and attitudes of the HCPs. Second, taking into account the staffs’ level of knowledge and skills, the APN used the following specific strategies to gain their support and to develop a level of trust, so they could turn to her when they had questions about working with persons with CI: (a) placed an emphasis on developing supportive relationships between APN and staff; (b) fostered an environment encouraging collaborative inquiry, where current practices were questioned in a thoughtful, respectful manner; (c) made the APN’s clinical supervision available to staff to provide point of care mentorship; (d) introduced staff to the principle of the dignity of risk when decisions about going home were difficult for the team; (e) provided reflective learning opportunities such that staff could articulate continuous insight into changing practice; and (f) attended weekly patient rounds to continue staff education on patient advocacy and to share their assessments. Management staff’s involvement included supporting staff during implementation of new knowledge into practice and meeting regularly with the APN on the unit to discuss any issues related to implementation.

To track treatment fidelity of the intervention implementation, several strategies were put into place as suggested by Kolanowski and colleagues [30]. Staff training at the different sites was conducted by the PI and the APN to ensure consistency in delivery. The APN kept a training log, including attendance record, for the workshop, informal bedside mentorship, and in-services provided to staff. Approximately 80% of all staff on both units participated in the workshop; for those staff who could not attend, the APN made an effort to provide additional mentoring on the unit. The APN conducted 18 in-services in each of the facilities over the one-year period on topics such as delirium, pain management, and incontinence with anywhere from 4 to 20 staff in attendance per in-service.

During implementation of the PCRM-CI, the APN kept a patient care log to ensure each patient received care reflective of the components of the model focused on them. Protocol adherence was maintained for 69 of the 73 patients. To improve protocol adherence, the APN made recommendations to the staff members in managerial positions on how to implement the model of care for each patient enrolled in the treatment cohort.

### Statistical analysis

Based on results from a pilot study [28], it was determined that a sample size of 39 patients for each group was required to detect a large effect size. In order to allow for attrition and to ensure that a sufficient number of persons with CI would be recruited, 70 patients were required in each group. Oversampling occurred to increase the number of persons with CI in each group. Univariate statistics were performed to describe the characteristics and outcomes of patients in the PCRM-CI intervention and usual care groups. Fisher’s exact test was used to test independence in contingency tables. Unpaired *t*-tests were used to assess differences between treatment group means.

Differences in FIMM scores by treatment group were tested using multivariate regression analyses that accounted for: (a) socio-demographic characteristics of age, gender, and living situation (i.e., living alone or with another person); (b) presence of comorbidities using the Charlson Comorbidities Index [31]; (c) length of stay in inpatient rehabilitation; (d) whether patients were cognitively impaired at admission (based on MMSE score); (e) admission FIMM score; (f) whether patients were cared for in the PCRM-CI or usual care group; and (g) the site of enrollment and care delivery.

## Results

One hundred sixty-three patients were eligible to participate; 14 declined because they did not want to involve a person to act as a collateral informant. Of the remaining eligible patients (N = 149), 20 were not competent to provide consent; in these cases, a substitute decision maker, who also acted as the patients’ collateral informant, consented on behalf of the patient. The usual care group included 76 patients, 23 with CI; the PCRM-CI group had 73 patients, 24 with CI. Information on 145 collateral informants was collected (four collateral informants agreed to participate but wished to exclude their demographics).

### Participant characteristics

Participant characteristics are summarized in Table 1. The majority of participants had an intracapsular or intertrochanteric hip fracture. Most participants had undergone surgery within one day of admission to inpatient rehabilitation and started active rehab within six days of surgery. More participants were female, 77% in the control group and 79% in the intervention group. The mean age did not differ between the usual care and intervention groups (mean age: 80.1 ± 6.7 and 82.5 ± 8.8, respectively; *p =* 0.06), although PCRM-CI intervention group included a greater proportion of older patients compared to the usual care group. The two groups were otherwise similar with regard to socio-demographic characteristics, overall health and functional status, and comorbidities. In both the usual care and PCRM-CI intervention groups, about one-third of participants had CI at admission as measured by MMSE (n = 23 or 30% and n = 24 or 33%, respectively). Most participants did not have delirium on admission nor did they develop delirium during their stay.

**Table 1:** Patient characteristics at admission

**Characteristic**	**Usual care, n = 76**	**Intervention, n = 73**	***P*-value**
Age			0.01
64–74	18 (24%)	18 (25%)	
65–84	37 (49%)	20 (27%)	
85+	21 (28%)	35 (48%)	
MMSE at admission			0.86
Impaired (MMSE < 24)	23 (30%)	24 (33%)	
Not impaired (MMSE ≥ 24)	53 (70%)	49 (67%)	
CAM at admission			0.24
No delirium	72 (95%)	65 (89%)	
Delirium	4 (5%)	8 (11%)	
Pre-fracture ambulation			0.61
Independent without gait aid	36 (47%)	36 (50%)	
Independent with gait aid	31 (41%)	32 (44%)	
Dependent with standby assistance	5 (7%)	3 (4%)	
Dependent with Min-Mod assistance	4 (5%)	2 (3%)	
Pre-fracture distance walked			0.69
> 1 block	47 (61%)	47 (65%)	
< 1 block	24 (32%)	22 (30%)	
Indoors only	5 (7%)	4 (5%)	
Pre-fracture history of falls with injury			0.86
Previous fall with injury	39 (56%)	38 (58%)	
No previous falls with injury	31 (44%)	28 (42%)	
Missing	6	7	
Comorbidities			0.20
0 to 2	9 (12%)	12 (16%)	
3	9 (12%)	17 (23%)	
4	14 (18%)	14 (19%)	
5	11 (14%)	10 (14%)	
6 or more	33 (43%)	20 (27%)	

SD – Standard Deviation; MMSE – Mini-Mental State Exam; CAM – Confusion Assessment Method; Min-Mod – minimum to moderate.

### Outcomes

Differences in patient outcomes between sites were compared to account for site differences in implementation of the PCRM-CI (Table 2). No differences in outcomes were found between the sites; subsequent analysis was performed on data pooled from both sites. FIMM scores did not differ significantly between the PCRM-CI intervention and usual care groups at admission, despite the intervention group having a greater proportion of older patients. Patients in both groups showed gains in mobility between admission and discharge (Table 2). Gains were negatively associated with length of stay and were lower for CI patients (Table 3). Gains were not greater in the PCRM-CI intervention group compared to the usual care group (Table 3). In contrast, patients in the PCRM-CI intervention group were more likely to return to home after discharge than patients who received usual care (Table 3). Living alone, length of stay, and CI were each negatively associated with the probability of returning home (Table 3).

**Table 2:** Patient outcomes: admission, discharge

**Outcome**	**Mean ± SD**		***p*-value**
	**Usual care, n = 76**	**Intervention, n = 73**	
FIMM at admission	13.9 ± 6.2	13.1 ± 5.4	0.37
FIMM at discharge	24.9 ± 6.4	24.3 ± 5.1	0.57
FIMM gain	10.9 ± 6.6	11.5 ± 5.7	0.58
Post-discharge living location, n (%)			0.07
Returned home	59 (79%)	64 (90%)	
Did not return home	16 (21%)	7 (10%)	
Missing data	1	2	

Note: Gain is defined as the difference in assessment scores between admission and discharge from inpatient rehabilitation; SD – Standard Deviation; FIMM – Functional Independence Measure Mobility subscale.

**Table 3:** Multivariate regression results for each patient outcome

**Variable**	**Coefficient (95% confidence interval)**		***p*-value**	
	**FIMM gain**	**Return to home**	**FIMM gain**	**Return to home**
FIMM admission score	−0.72 (−0.88 – -0.55)	–	<0.001	–
Pre-hip fracture OARS	–	0.12 (−0.00 – 0.24)	–	0.05
Age	−0.05 (−0.17 – 0.06)	−0.02 (−0.10 – 0.07)	0.38	0.71
Gender	−0.71 (−2.89 – 1.48)	1.01 (−0.57 – 2.60)	0.53	0.21
Pre-hip fracture living status	−0.50 (−2.37 – 1.36)	1.80 (0.22 – 3.37)	0.73	0.03
Charlson Comorbidity Index	0.07 (−0.48 – 0.62)	−0.08 (−0.32 – 0.48)	0.80	0.70
Length of stay	−0.12 (−0.20 – -0.05)	−0.05 (−0.08 – -0.02)	<0.001	<0.01
CI at admission	−2.38 (−4.31 – -0.46)	−1.52 (−2.87 – -0.17)	0.02	0.03
Site	0.55 (−1.21 – 2.32)	0.44 (0.75 – 1.63)	0.54	0.47
Intervention group	0.15 (−1.62 – 1.91)	1.45 (0.23 – 2.67)	0.87	0.02

FIMM – Functional Independence Measure Motor Subscale; CI – cognitive impairment as measured by MMSE; OARS – Older Americans Resources and Services Instrument.

## Discussion

Although the PCRM-CI intervention had components targeting older persons with CI and multiple comorbidities, the current study found no difference between the usual care and PCRM-CI groups in terms of mobility gains, as measured by FIMM. In retrospect, this finding is not surprising, considering that the FIMM is a standard rehabilitation tool mandated in Ontario through the National Rehabilitation Services and, in practice, is used as a measure of mobility independence and for purposes of determining if the patient can be discharged back home. As the intent of the current program was that patients would be discharged to home, FIMM discharge scores were similar for all patients in the usual care and PCRM-CI intervention groups. Differences were not seen in how soon patients reached similar FIMM scores between the two groups; however, the PCRM-CI intervention was not planned to accelerate recovery of mobility, and hence shorten length of stay, but to get patients back home. For future research, it is imperative that efforts be focused on determining the best outcome measure to use to evaluate whether the PCRM-CI intervention leads to meaningful functional recovery differences. Further, determining changes in staff attitudes and assumptions regarding rehabilitation care for persons with CI as a result of the PCRM-CI is another area for future work.

Patients assigned to the PCRM-CI intervention group were more likely to return to their prior residence after discharge than patients in the usual care group including those with CI. Before the PCRM-CI was introduced, when patients were confused or agitated, the healthcare team commonly discussed with family members the possibility of nursing home placement following rehabilitation. After the PCRM-CI was implemented, discharge plans were established close to the admission date, so that families were made aware of the expectation that the patient would return home, and staff developed individualized approaches when rehabilitating patients to try to achieve this goal. It is likely that these approaches, an awareness of the difference between delirium and dementia, and the expectations that the patient was returning to their pre-admission destination, influenced practice leading to more patients returning home. The success of such a model stems from staff knowing the person, adapting the social and physical environment as necessary, and relating effectively to gain patient trust, as outlined in the REAP model.

Building the knowledge base about best rehabilitation strategies (e.g., prompting, cueing, demonstrating) based on the person’s remaining cognitive abilities will be imperative as the population ages, patients become increasingly complex, and the prevalence of CI among the population increases. Given that direct health costs in Canada post-hip fracture range from $21,000 (for a patient discharged to home) to $47,000 (for a patient discharged to long-term nursing home care) [13], facilitating discharge to home would yield significant cost savings to the healthcare system. It is likely that other countries would similarly realize cost savings if home discharge rates could be improved.

This study had some limitations. First, there was no blinding of patients, collateral informants, or research assistants. Second, the limited sample size provided insufficient power to examine multiple outcomes and interactions among predictors. Third, this study used a quasi-experimental design as it was impossible to randomly assign the patients to the intervention or control groups which may have resulted in response bias. Finally, although the PCRM-CI was designed as a multicomponent intervention, the investigators did not include a precise method for capturing the dosage of each component of the intervention. This makes it difficult to determine the effects of the separate components. Future research will need to be focused on how to track fidelity when providing complex interventions by multiple clinicians on a unit over a 24-hour period for an average 18-day period.

## Conclusions

Demand is growing to evaluate the efficacy of interventions to improve recovery after hip fracture for older patients with complex comorbidities, including CI. The current study afforded preliminary evidence that providing additional education, support, and clinical resources (e.g., an APN with expertise in gerontology) in existing community rehabilitation units can increase the proportion of patients who return home post-discharge. While many patients with cognitive impairment continue to be denied access to inpatient rehabilitation post-hip fracture in many countries, implementing the PCRMI-CI is a viable option for enhancing access and care for those patients requiring active rehabilitation services post-hip fracture.

## Abbreviations

CI: Cognitive impairment; MSK: Musculoskeletal; HCP: Healthcare professional; PCRM-CI: Patient-centered rehabilitation model targeting older persons with a hip fracture, including those with cognitive impairment; APN: Advanced Practice Nurse; CAM: Confusion Assessment Method; MMSE: Mini-Mental State Exam; IQCODE: Informant Questionnaire on Cognitive Decline in the Elderly; OARS: Older Americans resource and services instrument; FIMM: Functional Independence Measure Motor Subscale.

## Competing interests

The authors’ declare that they have no competing interests.

## Authors’ contributions

KM, AD, SJ, GN, NM, JF, conceived the project and KM, AD, and SJ developed the hypotheses and the protocol. KM, SS, CC were responsible for data collection, management and data analyses. KM wrote the first draft of the article and all authors contributed to the development of the manuscript. All authors read and approved the final manuscript.

## Pre-publication history

The pre-publication history for this paper can be accessed here:

http://www.biomedcentral.com/1471-2318/13/136/prepub
